# Generation of live mice from haploid ESCs with germline-DMR deletions or switch

**DOI:** 10.1038/s41421-024-00757-x

**Published:** 2025-01-21

**Authors:** Yongjian Ma, Meng Yan, Zhenfei Xie, Hongling Zhang, Zhoujie Li, Yuanyuan Li, Suming Yang, Meiling Zhang, Wen Li, Jinsong Li

**Affiliations:** 1 Key Laboratory of Multi-Cell Systems, Shanghai Key Laboratory of Molecular Andrology, CAS Center for Excellence in Molecular Cell Science, Shanghai Institute of Biochemistry and Cell Biology, University of Chinese Academy of Sciences, Chinese Academy of Sciences, Shanghai, China; 2 Center for Reproductive Medicine & Fertility Preservation Program, International Peace Maternity and Child Health Hospital, School of Medicine, Shanghai Jiao Tong University, Shanghai, China; 3 Animal Core Facility, CAS Center for Excellence in Molecular Cell Science, Shanghai Institute of Biochemistry and Cell Biology, University of Chinese Academy of Sciences, Chinese Academy of Sciences, Shanghai, China; 4 School of Life Science and Technology, ShanghaiTech University, Shanghai, China

**Keywords:** Epigenetic memory, Embryonic stem cells, DNA methylation, Reprogramming

## Abstract

Genomic imprinting is required for sexual reproduction and embryonic development of mammals, in which, differentially methylated regions (DMRs) regulate the parent-specific monoallelic expression of imprinted genes. Numerous studies on imprinted genes have highlighted their critical roles in development. However, what imprinting network is essential for development is still unclear. Here, we establish a stepwise system to reconstruct a development-related imprinting network, in which diploid embryonic stem cells (ESCs) are derived by fusing between parthenogenetic (PG)- and androgenetic (AG)-haploid embryonic stem cells (haESCs) with different DMR deletions (termed Ha-Ha-fusion system), followed by tetraploid complementation to produce all-haESC fetuses. Diploid ESCs fused between PG-haESCs carrying 8 maternally-derived DMR deletions and AG-haESCs with 2 paternally-derived DMR deletions give rise to live pups efficiently, among which, one lives to weaning. Strikingly, diploid ESCs derived from the fusion of PG-haESCs with 7 maternal DMR deletions and AG-haESCs with 2 paternal DMR deletions and maternal *Snrpn*-DMR deletion also support full-term embryonic development. Moreover, embryos reconstructed by injection of AG-haESCs with hypomethylated *H19*-DMR into oocytes with *H19*-DMR deletion develop into live mice sustaining inverted allelic gene expression. Together, our findings indicate that restoration of monoallelic expression of 10 imprinted regions is adequate for the full-term development of all-haESC pups, and it works irrespective of their parental origins. Meanwhile, Ha-Ha-fusion system provides a useful tool for deciphering imprinting regulation networks during embryonic development.

## Introduction

In the 1980s, the bi-maternal and bi-paternal embryos were constructed by pronuclear transfer. These embryos exhibited developmental failure during mid-gestation, leading to the discovery of genomic imprinting^1–3^, as parental genomes bear distinct “marks” on their DNA. Genomic imprinting is an epigenetic process that causes a subset of genes to be monoallelically expressed in a parent-of-origin-specific manner. Most imprinted genes are organized in clusters, and their monoallelic expression is regulated by the parent-of-origin-specific acquisition of DNA methylation marks, termed the differentially methylated regions (DMRs)^4–7^. Genetic studies have demonstrated that DMR deletions could largely mimic their hypermethylated state in most loci, resulting in expected imprinting patterns during embryonic development^8–10^. Extensive research has highlighted the critical roles of imprinted genes during embryonic and fetal development^6–8^. However, the specific sets of paternal and maternal imprinted genes essential for development remain unclear, largely due to the lack of an efficient system to genetically manipulate imprinted control regions of both paternally and maternally imprinted loci.

Recently, mammalian haploid embryonic stem cells (haESCs) have been successfully created from blastocysts carrying only one set of maternal or paternal genome^11–16^. Notably, androgenetic-haESCs (AG-haESCs), derived from sperm-originated haploid blastocysts, could support full-term embryonic development when injected into oocytes, resulting in healthy animals, termed semi-cloned mice (SC mice)^12^. However, AG-haESCs gradually lost the potential to produce SC animals because two inhibitors of Mek1/2 and Gsk3β (2i) used in their culture medium induced a widespread loss of DNA methylation, including *H19-*DMR and IG-DMR^12,17^. Strikingly, AG-haESCs carrying both *H19*-DMR and IG-DMR deletions could efficiently support the development of SC embryos^18^, demonstrating that DMR deletions can simulate the paternal imprinting state. Similarly, parthenogenetic-haESCs (PG-haESCs), derived from haploid embryos carrying oocyte genomes and cultured in 2i medium, also lost DMR methylation over time, and this loss enabled these cells to produce SC mice after removal of both *H19*-DMR and IG-DMR^19,20^. Together, imprint-free haESCs with paternal DMR deletions can be used as sperm replacement to support embryonic development, implying that these cells with particular maternal DMR deletions may also be used as oocyte genome replacement to support embryonic development. We thus reasoned that replacing both sperm and oocyte genomes with haESC-derived DNA could offer a novel strategy to manipulate paternal and maternal imprints simultaneously, facilitating the identification of essential imprinting combinations for embryonic development. Moreover, this strategy might also enable systematic studies on interactions between paternal and maternal imprints.

In this study, we tested this concept by establishing a fusion system between AG-haESC and PG-haESC lines with parent-of-origin-specific-DMR edits (Ha-Ha-fusion system) to produce diploid ESCs carrying DMR-deletion combinations. These cells were then subjected to tetraploid (4N) embryo complementation to assess their developmental potential. The 4N embryo complementation method, widely utilized to rescue the placental dysfunction that otherwise would result in fetal death^21,22^, allows 4N cells to contribute extraembryonic tissues while rarely contributing to the embryo itself^23,24^. This strategy has been used to assess the pluripotency of ESCs and iPSCs, leading to the generation of all-ESC or all-iPSC mice^25,26^. Here, we demonstrated that a deletion combination of 10 regions, including paternal *H19*-DMR and IG-DMR, and maternal *Xist*-DMR, *Igf2r*-DMR, *Kcnq1*-DMR, *Gnas*-DMR, *Peg3*-DMR, *Nespas*-DMR, *Snrpn*-DMR, and *Grb10*-DMR could support reconstructed embryos to accomplish full-term embryonic development efficiently. Furthermore, we showed that the non-parent-of-origin-specific inheritance mode of DMR deletions could also support embryonic development, indicating that their primary functional role lies in regulating the dosage of imprinted genes rather than their parental origin.

## Results

### Establishment of Ha-Ha-fusion system based on hypomethylated haESCs induced by 2i

**Fig. 1:**
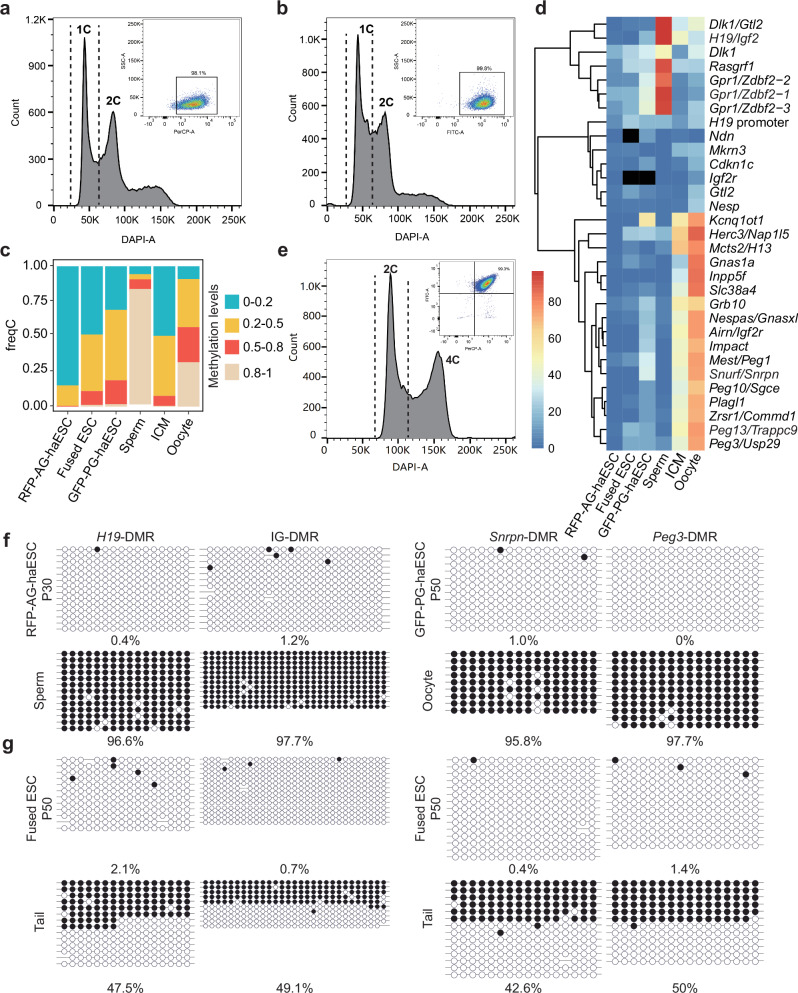
Establishment of the Ha-Ha-fusion system. **a** Establishment of RFP-labeled AG-haESCs by FACS enrichment for haploid cells. A DAPI filter was used to detect a signal of Hoechst 33342-stained DNA. A PerCP-A filter was used to detect a signal of RFP-positive cells (98.1%). **b** Establishment of GFP-labeled PG-haESCs by FACS enrichment for haploid cells. A DAPI filter was used to detect a signal of Hoechst 33342-stained DNA. A FITC-A filter was used to detect a signal of GFP-positive cells (99.8%). **c** Methylation levels of the whole genome in oocytes, sperm, inner cell mass (ICM), RFP-labeled AG-haESCs, GFP-labeled PG-haESCs and diploid fused ESCs. DNA methylome data of oocytes, sperm and ICM have been reported^31^. **d** Heatmap of imprinted region methylation in oocytes, sperm, ICM, RFP-labeled AG-haESCs, GFP-labeled PG-haESCs and diploid fused ESCs. Black squares indicate a lack of valid reads. **e** Establishment of diploid fused ESCs. A DAPI filter was used to detect a signal of Hoechst 33342-stained DNA. FITC-A filter and PerCP-A filter were used to detect a signal of double-positive cells (99.3%). **f** Bisulfite sequencing analysis of IG-DMR and *H19*-DMR in P30 RFP-labeled AG-haESCs and sperm, and *Snrpn*-DMR and *Peg3*-DMR in P50 GFP-labeled PG-haESCs and oocyte. Open and filled circles represent unmethylated and methylated CpG sites, respectively. **g** Bisulfite sequencing analysis of IG-DMR, *H19*-DMR, *Snrpn*-DMR and *Peg3*-DMR in P50 fused ESCs and tail. Open and filled circles represent unmethylated and methylated CpG sites, respectively. The passage number of fused ESCs is inherited from the passage number of PG-haESCs.

To comprehensively investigate the functional significance of DMRs in vivo, we aimed to establish a system capable of simultaneously manipulating maternal and paternal DMRs. To achieve this, we first derived RFP-labeled AG-haESCs (RFP-AG-haESCs) and GFP-labeled PG-haESCs (GFP-PG-haESCs) from *CAG-RFP* and *actin-EGFP* transgenic mice respectively^27,28^ (Supplementary Fig. S1a). The haESCs were then cultured in a standard ESC culture medium supplemented with 2i and maintained through FACS-enrichment of haploid cells regularly^12^ (Fig. 1a, b). Given that 2i induces global hypomethylation following passaging, as reported previously^17,29,30^, whole-genome bisulfite sequencing (WGBS) on GFP-PG-haESCs and RFP-AG-haESCs at passage 30 (P30) was performed. This analysis revealed that both haploid cell lines exhibited global hypomethylation compared to oocyte and sperm^31^ (Fig. 1c; Supplementary Fig. S1b–g). Notably, all paternal DMRs and the vast majority of maternal DMRs were hypomethylated (Fig. 1d). Subsequently, RFP-AG-haESCs and GFP-PG-haESCs were fused using HVJ virus, and the diploid (2N) fused cells with both GFP and RFP signals were enriched by FACS (termed Ha-Ha-fusion system, Fig. 1e). To assess their imprinted state, we analyzed the methylation state of 2N ESCs fused using PG-haESCs at P50 and found that the fused ESCs exhibited DNA methylation levels between AG-haESCs and PG-haESCs (Fig. 1c; Supplementary Fig. S1b–g) and sustained hypomethylated state at imprinted regions (Fig. 1d). We further investigated the methylation status of DMRs in haploid ESCs and 2N fused cells across different passages using bisulfite PCR. Consistently, RFP-AG-haESCs (P30) displayed hypomethylation at *H19*-DMR and IG-DMR (Fig. 1f), and GFP-PG-haESCs (P30) retained methylation at a reduced level at *Snrpn*-DMR and *Peg3*-DMR (Supplementary Fig. S1h). However, by P50, GFP-PG-haESCs lost all DNA methylation at *Snrpn*-DMR and *Peg3*-DMR (Fig. 1f). Similarly, 2N fused ESCs showed a near-complete loss of DNA methylation at two tested DMRs by P50 (Fig. 1g). Together, these findings indicate that haESCs and 2N fused ESCs exhibit imprinted-free state after long-term culture under a condition with 2i.

### Embryos reconstructed from Ha-Ha-fusion-derived diploid ESCs with paternal DKO and six maternal DMR deletions develop to E15.5

**Fig. 2:**
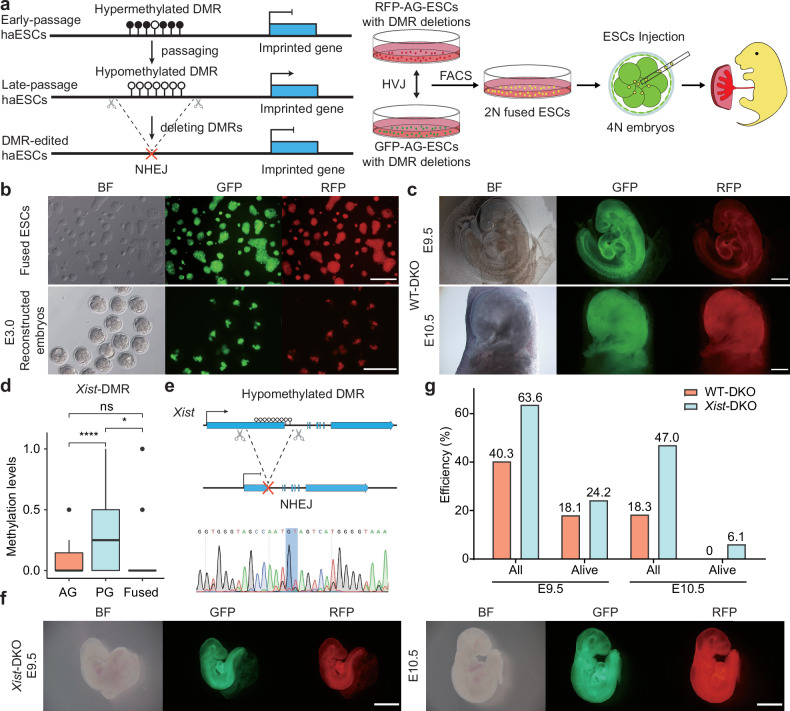
Generation of embryos with parental-allele-specific DMR deletions from ESCs derived by Ha-Ha-fusion system. **a** Scheme of generating embryos with parental-allele-specific DMR deletions. Hypomethylated DMRs were deleted by CRISPR-Cas9 in RFP-labeled AG-haESCs and GFP-labeled PG-haESCs. Diploid fused cells were obtained by fusing RFP-labeled AG-haESCs with GFP-labeled PG-haESCs with HVJ virus. 2N double-positive cells were enriched by FACS. Reconstructed embryos were generated by tetraploid complementation of fused cells. **b** Colony morphology and fluorescence labeling of diploid fused ESCs (top) and E3.0 reconstructed embryos (bottom). Scale bars, 200 µm. BF, bright field. **c** E9.5 (top) and E10.5 (bottom) reconstructed embryos (WT-DKO) carrying paternal *H19*-DMR and IG-DMR deletions. WT-DKO embryos were generated from late passages (P30) fused ESCs. The passage number of fused ESCs is inherited from the passage number of PG-haESCs. Scale bars, 500 µm. **d** DNA methylation levels at *Xist*-DMR in AG-haESCs, PG-haESCs and fused ESCs. Data are presented as mean ± SD, analyzed by Student’s *t*-test, *P < 0.05, ****P < 0.0001, ns, not significant. **e** Top, diagram showing deletion of *Xist*-DMR. Bottom, DNA sequences of PCR products amplified from *Xist*-DMR’s deleted region in *Xist*-DMR deletion PG-haESCs. **f** E9.5 (left) and E10.5 (right) reconstructed embryos (*Xist*-DKO) carrying paternal DKO and maternal *Xist*-DMR deletion. Scale bars, 1 mm. **g** A comparison of the developmental (the number of embryos in the amniotic sac divided by the number of embryos transferred) and alive rates of reconstructed embryos in **c** and **f**. Alive status is judged by the heartbeat. Data were generated from Table 1.

We next investigated whether all-haESC mice can be generated through injection of 2N ESCs fused between RFP-AG-haESCs and GFP-PG-haESCs with DMR deletions into tetraploid embryos (Fig. 2a). To this end, *H19*-DMR and IG-DMR were deleted in RFP-AG-haESCs (termed RFP-DKO) to mimic paternal inherited imprints (Supplementary Fig. S2a). To evaluate the feasibility of the Ha-Ha-fusion system, wild-type GFP-PG-haESCs (GFP-WT) at different passages were fused with RFP-DKO cells. The resulting 2N fused ESCs (WT-DKO) carrying both GFP and RFP markers were enriched by FACS and their developmental potential was assessed through tetraploid complementation (Fig. 2b). After transplantation of reconstructed embryos into uteruses of recipient females, WT-DKO cells (P8) derived from early-passage GFP-WT cells (P5) could support full-term embryonic development with a birth rate at around 15%, half of which survived to adulthood (Table 1; Supplementary Fig. S2b, c). By contrast, the reconstructed embryos derived from WT-DKO cells (P30) fused between RFP-DKO and late-passage GFP-WT cells (P27) were completely arrested at E10.5 (Fig. 2c). These results highlight the importance of maternal imprints, as their absence results in developmental failure of reconstructed embryos before E10.5.

**Table 1:** Summary of reconstructed embryos obtained from Ha-Ha-fusion-derived ESCs.

2N fused ESC line*	No. of Transferred Embryos (TE)	No. of Observed Embryos (% TE)	No. of Alive Embryos (% TE)	No. of Transferred Embryos (TE)	No. of Observed Embryos (% TE)	No. of Alive Embryos (% TE)	No. of Transferred Embryos (TE)	No. of Observed Embryos (% TE)	No. of Alive Embryos (% TE)
Embryo stage	E9.5			E10.5			E11.5		
WT-DKO P30	72	29 (40.3)	13 (18.1)	60	11 (18.3)	0	ND^a^	ND	ND
*Xist*-DKO	33	21 (63.6)	8 (24.2)	66	31 (47.0)	4 (6.1)	ND	ND	ND
XI-DKO	ND	ND	ND	34	13 (38.2)	10 (29.4)	34	8 (23.5)	6 (17.6)
Embryo stage	E13.5			E14.5			E15.5		
XI-DKO	32	4 (12.5)	0	ND	ND	ND	ND	ND	ND
XIK-DKO	168	15 (8.9)	6 (3.6)	76	2 (2.6)	0	ND	ND	ND
XIKNP-DKO	87	7 (8.0)	3 (3.4)	120	19 (15.8)	3 (2.5)	52	7 (13.5)	0
XIKGPN-DKO	35	3 (8.6)	2 (5.7)	110	2 (1.8)	2 (1.8)	96	4 (4.2)	1 (1.0)
Embryo stage	E17.5			Full-Term					
XIKGPN -DKO	90	8 (8.9)	0	ND	ND	ND			
7KO-DKO	16	2 (12.5)	2 (12.5)	136	10 (7.4)	7 (5.1)			
8KO-DKO	ND	ND	ND	322	37 (11.5)	24 (7.5)^b^			
WT-DKO P8	34	7 (20.6)	7 (20.6)	51	8 (15.7)	8 (15.7)^c^			

^a^ Not determined.

^b^ Two pups survived over 1 week (one died at P8 and the other one died at P23).

^c^ 4 pups survived to adulthood.

^*^ Injection data of each cell line were collected from over 2 cell lines. To avoid the potential side effects caused by CRISPR-Cas9-mediated DMR deletions, we excluded the extreme values of developmental rate from the cell lines that did not induce implantation or caused abnormal development of all reconstructed embryos.

To identify maternal DMRs critical for embryonic development, we sought to enhance the developmental potential of reconstructed embryos by deleting maternal DMRs in GFP-PG-haESCs. Previous studies on somatic cell nuclear transfer (SCNT)-derived cloned embryos indicated that ectopic expression of maternal *Xist* contributed to implantation defects^32^. Reducing *Xist* dosage, either by using *Xist* heterozygous knockout donor cells or injecting small interfering RNA (siRNA) against *Xist* into one-cell cloned embryos, significantly increased cloning efficiency by 8- to 9-fold^32,33^. Given that haESCs carry only X chromosome^12^ and 2N fused ESCs are female cells with XX (Supplementary Fig. S2d), we thus reanalyzed WGBS results and found that 2N fused ESCs completely lost all DNA methylation at the *Xist* promotor (DMR) (Fig. 2d), implying that aberrant X-chromosome inactivation (XCI) may be involved in developmental failure of reconstructed embryos from 2N WT-DKO ESCs. To address this, we deleted *Xist*-DMR in GFP-WT cells (termed GFP-*Xist*-KO) (Fig. 2e), and then fused them with RFP-DKO cells to produce a stable diploid cell line (termed *Xist*-DKO). Tetraploid complementation experiments revealed that *Xist*-DMR deletion in GFP-WT dramatically improved post-implantation developmental rate of *Xist*-DKO embryos at E10.5 (47.0% compared to 18.3% of WT-DKO embryos, Table 1). Moreover, 6.1% of transferred embryos remained viable at E10.5 (Fig. 2f, g). These results indicate that correction of *Xist* dosage can dramatically rescue embryonic development of the reconstructed embryos.

To further improve the developmental potential of reconstructed embryos, we targeted additional maternal loci essential during mid-gestation by deleting *Igf2r-* and *Kcnq1*-DMRs in GFP-*Xist*-KO cells, whose deletion caused embryonic lethality before E15^34–37^. The resultant cells (termed GFP-XIK) were fused with RFP-DKO cells to produce diploid cells carrying five DMR deletions (termed XIK-DKO) (Supplementary Fig. S2e), which could develop to E13.5 through tetraploid complementation (Table 1; Supplementary Fig. S2f, g), but all arrested at E14.5 (Table 1; Supplementary Fig. S2g).

To restore maternally imprinted states critical for late gestation, we deleted additional DMRs in GFP-XIK cells. Previous studies showed that imprinting abnormalities in *Nespas*/*Gnas* locus led to developmental deficiency at late gestation^38–42^, while *Peg3* locus modulates fetal growth at late gestation^43^ and its aberrant expression causes partial embryonic lethality and reduced body weight^44^. Thus, *Gnas*-DMR and *Peg3*-DMR were deleted in the cell line GFP-XIK (termed GFP-XIKGP) (Supplementary Fig. S2e). In vivo developmental potency analysis indicated that GFP-XIKGP cells, when fused with RFP-DKO cells (XIKGP-DKO), extended embryonic development to E14.5 (2.5%) (Table 1; Supplementary Fig. S2h, i). Furthermore, after removal of *Nespas*-DMR in GFP-XIKGP cells to produce a cell line termed GFP-XIKGPN (Supplementary Fig. S2e), we found that the reconstructed embryos (XIKGPN-DKO) could further develop to E15.5 (Supplementary Fig. S2j), though no embryos (0/90) survived to E17.5 (Table 1; Supplementary Fig. S2j, k). In conclusion, the deletion of 6 maternal DMRs critical for embryonic development in PG-haESCs improved the developmental potential of reconstructed embryos, extending their viability from E9.5 to E15.5.

### Efficient generation of live pups using Ha-Ha-fusion-derived diploid ESCs with two more maternal DMR deletions

**Fig. 3:**
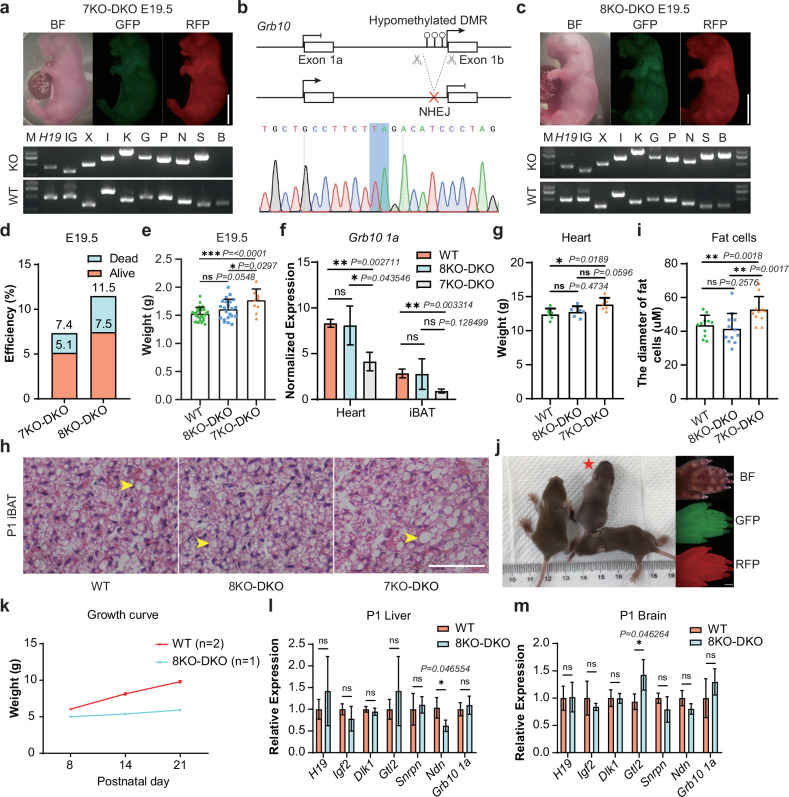
Efficient generation of alive mice from ESCs with 8 maternal DMR deletions and 2 paternal DMR deletions (8KO-DKO). **a** Top, E19.5 reconstructed embryos (7KO-DKO) carrying paternal DKO and maternal 7KO. 7 maternal imprinted regions are *Xist* (X), *Igf2r* (I), *Kcnq1* (K), *Gnas* (G), *Peg3* (P), *Nespas* (N) and *Snrpn* (S). Bottom, Genotyping of the 7KO-DKO ESCs with PCR. Genotypes of the deletion and WT were checked for paternal DKO and maternal 7KO. Scale bar, 5 mm. **b** Top, diagram showing deletion of *Grb10*-DMR. Bottom, DNA sequences of PCR products amplified from *Grb10*-DMR’s deleted region in GPF-XIKGPNSB PG-haESCs. **c** Top, E19.5 reconstructed embryos (8KO-DKO) carrying paternal DKO and maternal 8KO. 8 maternal regions are 7 maternal regions in **a** and *Grb10*-DMR (B). Bottom, genotyping of the 8KO-DKO ESCs with PCR. Genotypes of the deletion and WT were checked for paternal DKO and maternal 8KO. Scale bar, 5 mm. **d** Development rate of 7KO-DKO and 8KO-DKO embryos at E19.5. Alive status is judged by the breath. Data were generated from Table 1. **e** The body weights of E19.5 BDF1 (WT, *n* = 30), 8KO-DKO (*n* = 22) and 7KO-DKO (*n* = 10) embryos. Data are presented as mean ± SD, analyzed by Student’s *t*-test, *P < 0.05, ***P < 0.001, ns, not significant. **f** Expression analysis of *Grb10 1a* in hearts and iBATs of P1 WT, 8KO-DKO and 7KO-DKO pups (*n* = 3 for each group). Data are presented as mean ± SD, analyzed by Student’s *t*-test, *P < 0.05, **P < 0.01, ns, not significant. **g** The weights of heart from P1 WT (*n* = 6), 8KO-DKO (*n* = 6) and 7KO-DKO (*n* = 6) pups. Data are presented as mean ± SD, analyzed by Student’s *t*-test, *P < 0.05, ns, not significant. **h** H&E staining of iBATs from P1 WT, 7KO-DKO and 8KO-DKO mice. Scale bar, 400 µm. Multilocular lipid droplets were marked by yellow arrows. **i** Average diameters of fat cells (*n* = 12) from iBATs in P1 WT, 8KO-DKO and 7KO-DKO pups. Data are presented as mean ± SD, analyzed by Student’s *t*-test, **P < 0.01, ns, not significant. **j** Left, postnatal 23 day mice of WT and 8KO-DKO. 8KO-DKO pup was marked by a red asterisk. Right, fluorescent images of 8KO-DKO pup’s forepaw. Scale bar, 5 mm. **k** The growth curve of WT (*n* = 2) and 8KO-DKO (*n* = 1) mice. **l, m** Expression analysis of imprinted genes (*H19*, *Igf2*, *Dlk1*, *Gtl2*, *Snrpn*, *Ndn* and *Grb10* 1a) in liver (**l**) and brain (**m**) of P1 WT and 8KO-DKO pups (*n* = 3 for each group). Data are presented as mean ± SD, analyzed by Student’s *t*-test, *P < 0.05, ns, not significant.

A recent study showed that *Snrpn*-DMR and *Grb10*-DMR deletions contribute to the full-term development of bi-paternal embryos^45^. *Snrpn* locus is associated with prenatal growth, and its abnormalities lead to multiple postnatal phenotypes, including neonatal hypotonia and failure to thrive^46–48^. To investigate its role, we first deleted *Snrpn*-DMR in the GFP-XIKGPN cells to produce a cell line with 7 maternal DMR deletions (termed GFP-XIKGPNS) (Supplementary Fig. S2e, l). Following fusion with RFP-DKO cells, 4N complementation analysis showed that 5.1% of the resultant reconstructed embryos (termed 7KO-DKO) were viable at E19.5 (Fig. 3a and Table 1; Supplementary Fig. S2m). However, all pups died shortly after birth. These findings indicate that the re-establishment of maternal *Snrpn*-DMR enables full-term development of reconstructed embryos, suggesting a critical role of *Snrpn*-imprinted region during perinatal stages.

Next, we targeted the *Grb10* locus, which is associated with lipid biogenesis and regulation of social behaviors^49,50^. We deleted *Grb10*-DMR in the GFP-XIKGPNS cells to produce GPF-XIKGPNSB cells (Fig. 3b; Supplementary Fig. S2n). Remarkably, 11.5% (37/322) of the resultant reconstructed embryos (termed 8KO-DKO) survived to E19.5 (Fig. 3c, d), with 64.9% (24/37) of the embryos remaining viable (Fig. 3d and Table 1). Interestingly, the body weights of 8KO-DKO-originated pups were lighter than those of 7KO-DKO pups but similar to WT (BDF1) female pups (Fig. 3e). These results indicate that the deletion of *Grb10*-DMR mitigates the overgrowth of 7KO-DKO embryos, coincident with a previous study showing that *Grb10 1a* expression inhibited growth^51^. Accordingly, the *Grb10 1a* expression was up-regulated in tested tissues of 8KO-DKO mice compared to that in 7KO-DKO embryos (Fig. 3f), suggesting that *Grb10*-DMR deletion effectively simulates the methylated allele. Further analysis revealed that the hearts of 7KO-DKO mice were heavier than those of the WT mice, whereas no significant differences in heart weight were observed between 8KO-DKO and WT pups (Fig. 3g; Supplementary Fig. S3a, b). Although the weights of interscapular brown adipose tissue (iBAT) were comparable across groups, histological analysis using hematoxylin and eosin (H&E) staining showed larger multilocular lipid droplets in iBAT of the 7KO-DKO pups at postnatal day 1 compared to the WT and 8KO-DKO mice (Fig. 3h, i; Supplementary Fig. S3c). These results are consistent with previous observations that *Grb10 1a* is critical for the development of the heart and iBAT^50,52^.

While normal-weight pups were successfully fostered, most died within a few days of birth (Fig. 3j; Supplementary Fig. S3d, e). Two pups survived over one week; one of the pups that survived displayed obvious growth retardation and died at postnatal day 8 (Supplementary Fig. S3e, f), while the other failed to thrive and died at postnatal day 23 (Fig. 3j, k). Gene expression analysis revealed that although DMR deletions partially rescued the expression of genes located in these imprinted regions, several critical genes remained aberrantly expressed during development (Fig. 3l, m; Supplementary Fig. S3g, h). Furthermore, WGBS analysis of tail samples from postnatal day 23 8KO-DKO mice (D23) and WT mice (BDF1) showed that the majority of DMRs, including all deleted regions, were hypomethylated in the D23 sample (Supplementary Fig. S4a, b). However, secondary DMRs sustain a high methylation level in the D23 sample, similar to that in the WT sample (Supplementary Fig. S4c, d), suggesting that the deletion of germline DMRs can induce the establishment of the DNA methylation status in secondary DMRs during development. Taken together, while DMR deletions do not fully restore normal expression patterns of imprinted gene clusters, parental-allele-specific deletion of 10 DMRs can efficiently support the full-term development of reconstructed embryos.

### Embryos reconstructed from Ha-Ha-fusion-derived diploid ESCs with paternally-derived *Snrpn*-DMR deletion can develop to term

**Fig. 4:**
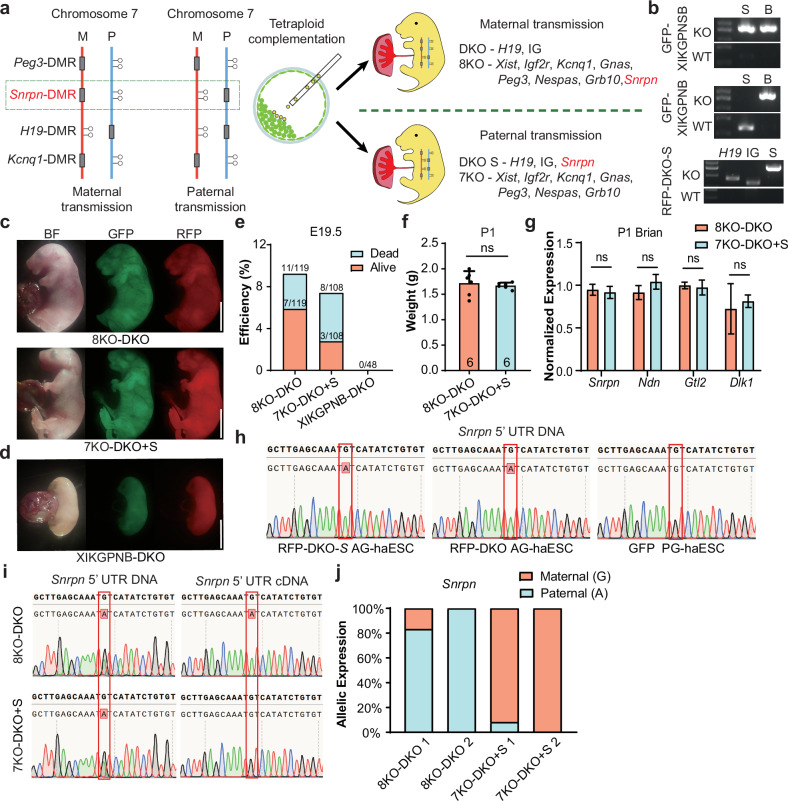
Paternal transmission of *Snrpn*-DMR deletion supports full-term development of reconstructed embryos. **a** Schematic diagram showing the generation of reconstructed embryos (7KO-DKO + S) carrying paternally derived *Snrpn*-DMR deletion. *Snrpn*-DMR was deleted in RFP-DKO AG-haESCs (termed RFP-DKO-S), and *Xist*-DMR, *Igf2r*-DMR, *Kcnq1*-DMR, *Gnas*-DMR, *Peg3*-DMR, *Nespas*-DMR and *Grb10*-DMR were deleted in PG-haESCs (termed GFP-XIKGPNB). **b** Genotyping of the related cell lines with PCR. Genotypes of GFP-XIKGPNSB PG-haESCs, GFP-XIKGPNB PG-haESCs and RFP-DKO-S AG-haESCs were indicated on the top, middle and bottom gel images, respectively. **c** Left, fluorescent images of E19.5 8KO-DKO pups. Right, fluorescent images of E19.5 7KO-DKO + S pups. Scale bars, 5 mm. **d** Fluorescent images of E17.5 XIKGPNB-DKO embryos. Scale bar, 5 mm. **e** Development rate of 8KO-DKO, 7KO-DKO + S and XIKGPNB-DKO embryos at E19.5. Alive status is judged by the breath. **f** The body weights of P1 8KO-DKO (*n* = 6) and 7KO-DKO + S pups (*n* = 6). Data are presented as mean ± SD, analyzed by Student’s *t*-test, ns, not significant. **g** Expression analysis of *Snrpn*, *Ndn*, *Gtl2* and *Dlk1* in brain of E19.5 8KO-DKO and 7KO-DKO + S embryos (*n* = 3 for each group). Data are presented as mean ± SD, analyzed by Student’s *t*-test, ns, not significant. **h** *Snrpn* 5’ UTR DNA sequences of PCR products amplified from DNA of RFP-DKO-S AG-haESCs (top), RFP-DKO AG-haESCs (middle) and PG-haESCs (bottom). **i** Left, *Snrpn* 5’ UTR DNA sequences of PCR products amplified from tail DNA of 8KO-DKO (top) and 7KO-DKO + S (bottom) embryos. Right, *Snrpn* 5’ UTR DNA sequences of PCR products amplified from brain cDNA of 8KO-DKO (top) and 7KO-DKO + S (bottom) embryos. **j** The relative allelic expression of the *Snrpn*, as measured by *Snrpn* 5’ UTR SNP analysis from brains cDNA of 8KO-DKO and 7KO-DKO + S embryos.

Having demonstrated that DMR-deletion combinations in haploid cells can mimic parental imprinting state and support full-term embryonic development, we next investigated whether parental origins of germline DMRs would affect their function on development by changing the location of *Snrpn*-DMR deletion. To this end, we deleted the *Snrpn*-DMR in the RFP-DKO cells to mimic paternally-derived *Snrpn* imprinting (termed RFP-DKO-S) and fused these cells with GFP-XIKGPNB cells (Fig. 4a). Remarkably, 4N complementation analysis showed that the resultant cells (termed 7KO-DKO + S) supported full-term embryonic development achieving a developmental state comparable to that of fused cells with maternally derived *Snrpn*-DMR deletion (8KO-DKO) (Fig. 4b, c). By contrast, XIKGPNB-DKO embryos generated from fused cells between RFP-DKO and GFP-XIKGPNB exhibited growth-retarded phenotype at E17.5, highlighting the critical role of paternal *Snrpn*-DMR deletion in enabling full-term development of reconstructed embryos (Fig. 4d, e). Additionally, embryos derived from 7KO-DKO + S cells had similar body weights to 8KO-DKO embryos (Fig. 4f). These results indicated that paternal transmission of *Snrpn*-DMR deletion extends the developmental potential of fused cells lacking *Snrpn*-DMR deletion from E17.5 to birth.

To further validate these results, we analyzed the expression patterns of the typical genes in the brain, where both IG-DMR and *Snrpn*-DMR play vital roles during the perinatal development stage. The expression levels of all tested genes in both imprinted clusters were comparable between 7KO-DKO + S mice and 8KO-DKO mice (Fig. 4g). Since the single nucleotide polymorphisms (SNPs) lack in *Snrpn* transcripts between RFP-AG-haESCs and GFP-PG-haESCs, to analyze the parental origins of gene expression, we introduced an adenine to replace guanine in 5’ LTR of *Snrpn* in RFP-AG-haESCs to distinguish its expression from maternal expression of GPF-PG-haESCs carrying the guanine at the same site (Fig. 4h). As expected, *Snrpn* was expressed from the paternal allele in the brain of 8KO-DKO mice as a maternally imprinted gene and expressed from maternal allele in 7KO-DKO + S mice as a paternally imprinted gene (Fig. 4i, j). Together, these results show that the maternally or paternally-derived *Snrpn*-DMR deletion functions equivalently during embryonic development of reconstructed embryos, suggesting that dosage regulation, rather than parental origin, is the key of imprinted genes.

### SC embryos with maternally-derived *H19*-DMR deletion can develop into adults with an inverted expression of the imprinted gene

**Fig. 5:**
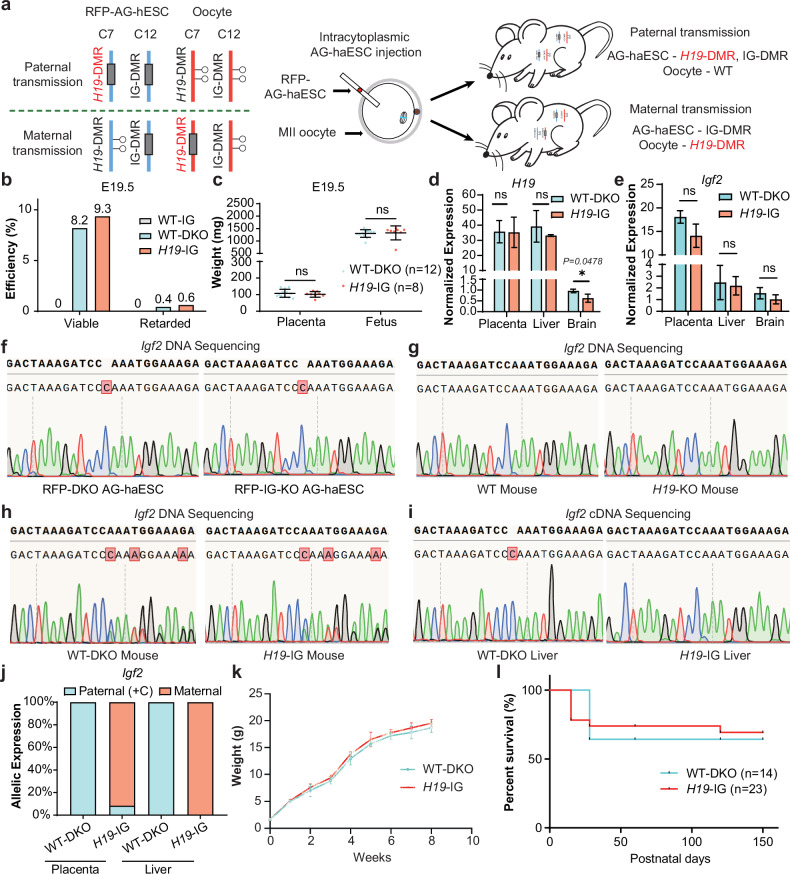
SC mice with maternal transmission of *H19*-DMR deletion grow normally to adult. **a** Schematic diagram showing generation of SC embryos (*H19*-IG embryos) carrying maternally-derived *H19*-DMR deletion. *H19*-DMR was deleted in oocytes, which derived from *H19*-KO mice. IG-DMR was deleted in RFP AG-haESCs (RFP-IG-KO). Reconstructed embryos were obtained by intracytoplasmic AG-haESCs injection (ICAHCI). **b** A comparison of the development rates of E19.5 WT-IG, WT-DKO and *H19*-IG embryos. Viable status is judged by the breath. Data were generated from Table 2. **c** Fetal and placental weights of E19.5 WT-DKO (*n* = 12) and *H19*-IG (*n* = 8) embryos. Data are presented as mean ± SD, analyzed by Student’s *t*-test, ns, not significant. **d**, **e** Expression analysis of *H19* (**d**) and *Igf2* (**e**) in placenta, liver and brain of P1 WT-DKO and *H19*-IG pups (*n* = 3 for each group). Data are presented as mean ± SD, analyzed by Student’s *t*-test, *P < 0.05, ns, not significant. **f–i** *Igf2* 5’ UTR DNA sequences of PCR products amplified from DNA of RFP-DKO and RFP-IG-KO AG-haESCs (**f**), tail DNA of WT and *H19*-KO mouse (**g**), tail DNA of WT-DKO and *H19*-IG mouse (**h**), liver cDNA of WT-DKO and *H19*-IG mouse (**i**). **j** Relative allelic expression of the *Igf2*, as measured by *Igf2* 5’ UTR SNP analysis from placenta and liver cDNA of WT-DKO and *H19*-IG pups. **k** Growth curve of WT-DKO (*n* = 5) and *H19*-IG (*n* = 5) mice. **l** Survival curve of WT-DKO (*n* = 14) and *H19*-IG (*n* = 23) mice.

We next investigated whether paternally methylated *H19*-DMR could also function properly through maternal transmission. Our previous studies have shown that the majority of SC embryos generated by injection of WT AG-haESCs into oocytes exhibited growth-retarded phenotypes due to loss of *H19*-DMR and IG-DMR methylation^12^. Furthermore, it was shown that deletion of both *H19*-DMR and IG-DMR in AG-haESCs (DKO-AG-haESCs) enabled efficient support of full-term SC embryo development^18^. Based on this, we tested whether SC embryos generated by injection of AG-haESCs with IG-DMR deletion into oocytes with *H19*-DMR deletion could develop into viable animals. To achieve this, we first generated a mouse line carrying homozygous *H19*-DMR deletions (termed *H19*-KO mice) through breeding SC mice generated from DKO-AG-haESCs. Subsequently, we deleted IG-DMR in the hypomethylated RFP-AG-haESCs (termed RFP-IG-KO AG-haESCs) (Fig. 1f). Next, RFP-IG-KO AG-haESCs were injected into mature oocytes with *H19*-DMR deletion to produce SC embryos with paternally-derived IG-DMR deletion and maternally-derived *H19*-DMR deletion (termed *H19*-IG embryos) (Fig. 5a; Supplementary Fig. S5a). SC embryos were collected at E12.5 to assess development, as a previous study highlighted the critical role of *H19*-DMR during early embryogenesis^53^. The results showed that *H19*-IG embryos exhibited developmental features comparable to those of SC embryos obtained by injection of RFP-DKO-AG-haESCs into WT oocytes (WT-DKO embryos) (Table 2; Supplementary Fig. S5b–d). By contrast, SC embryos derived from the injection of AG-haESCs with only IG-DMR deletion into WT oocytes (WT-IG embryos) were degenerative at E12.5^53^. These results indicated that maternal deletion of *H19*-DMR plays critical roles for the development of *H19*-IG embryos at E12.5. Consistent with this, the expression levels of genes located in this imprinted locus were overall similar between the fetus and placenta of E12.5 *H19*-IG embryos and E12.5 WT-DKO embryos (Supplementary Fig. S5e–h). However, *H19* expression was lower in the fetus of *H19*-IG embryos (Supplementary Fig. S5e), potentially due to residual *H19*-DMR methylation in some SC embryos^30^. Moreover, we dissected *H19*-IG embryos at E16.5 and found that they also displayed developmental characteristics as WT-DKO embryos (Table 2; Supplementary Fig. S5i–n).

**Table 2:** In vivo developmental of SC embryos.

Embryo Stage	Oocyte Genotype	AG-haESC Line	No. of Transferred Embryos (TE)	No. of Retarded Embryos (% TE)	No. of Normal Embryos (% TE)
E12.5	WT	*H19*-DMR and IG-DMR deletions	120	2 (1.7)	15 (12.5)
	*H19*-DMR deletion	IG-DMR deletion	80	0	9 (11.3)
E16.5	WT	*H19*-DMR and IG-DMR deletions	200	0	17 (8.5)
	*H19*-DMR deletion	IG-DMR deletion	220	2 (0.9)	17 (7.7)
Full-Term	WT	*H19*-DMR and IG-DMR deletions	232	1 (0.4)	19 (8.2)
	*H19*-DMR deletion	IG-DMR deletion	492	3 (0.6)	46 (9.3)
	WT	IG-DMR deletion	156	0	0

Notably, 46 alive *H19*-IG mice were produced from 492 transferred embryos, achieving a developmental efficiency of 9.3%, similar to that of WT-DKO mice (8.2%, 19/232, Fig. 5b and Table 2; Supplementary Fig. S5o). Consistently, they had similar weights and gene expressions as WT-DKO pups (Fig. 5c–e). To confirm the parental origin of transcripts, we introduced one cytosine in 5'-LTR of *Igf2* into RFP AG-haESCs (Fig. 5f, g). As expected, the resultant *H19*-IG embryos expressed maternally-biased expressed *Igf2* in all tested tissues (Fig. 5h–j). Moreover, *H19*-IG mice showed normal postnatal growth (Fig. 5k, l). These findings demonstrate that maternal transmission of *H19*-DMR deletion could support the normal development of SC embryos. Collectively, our findings indicate that non-parent-of-origin-specific inheritance mode of DMRs is capable of supporting embryonic development normally, implying that the primary function of DMRs is to regulate the expression dosage of imprinted genes rather than their parental origin.

## Discussion

Parental-origin-specific genomic imprinting is critical for mammalian development^6,7^. However, systematic studies on the functional significance of imprinting networks have been limited due to the absence of an efficient strategy to simultaneously manipulate both maternal and paternal imprints. In this study, we showed that the combined application of Ha-Ha-fusion and 4N embryo complementation methods allows a stepwise restoration of parental imprints for embryonic development, enabling the generation of live all-haESC-derived pups. These results highlight that 10 imprinted clusters, including two paternally imprinted loci, *H19*-*Igf2* and *Dio3*-*Dlk1*, and eight maternally imprinted loci, *Xist*, *Airn*-*Igf2r*, *Kcnq1*, *Gnas*, *Peg3*, *Nespas*-*Gnasxl*, *Snrpn*, and *Grb10*, are sufficient for full-term embryonic development. Our results confirm that monoallelic expression of imprinted genes is essential for embryonic and fetal development. Additionally, we showed that germline DMR deletions can be switched between the paternal and maternal genome, emphasizing that it is the monoallelic expression of imprinted genes, rather than their parental origin, that is crucial for proper development and growth.

**Fig. 6:**
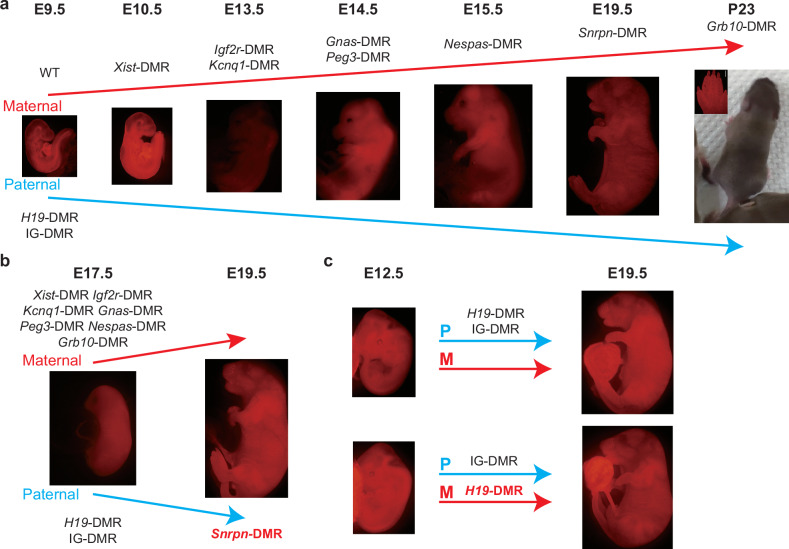
Schematic diagram of reconstructed embryos generated from Ha-Ha-fusion system and SC system. **a** Schematic diagram showing generation of reconstructed embryos carrying 8 maternal DMR deletions and 2 paternal DMR deletions. Developmental potential of reconstructed embryos was improved from E9.5 to postnatal day 23. **b,** **c** Schematic diagram showing that *Snrpn*-DMR (**b**) and *H19*-DMR (**c**) with inverse parental origins can regulate proper embryonic growth.

Here, we fixed the paternal imprinted state using AG-haESCs with both *H19*-DMR and IG-DMR deletions and investigated the appropriate maternal imprinted state for embryonic development using PG-haESCs with different maternal DMR deletions. Through a stepwise strategy, we systematically analyzed the relationship between maternal DMRs and embryonic development. Our results indicate that maternal deletion of *Xist*-DMR can improve the developmental rate of reconstructed embryos without maternal DMR deletions (WT-DKO) from E9.5 to E10.5; *Igf2r*-DMR and *Kcnq1*-DMR deletions can improve the developmental potential to E13.5; *Gnas*-DMR and *Peg3*-DMR deletions can improve the developmental potential to E14.5; *Nespas*-DMR deletion can improve the developmental potential to E15.5; *Snrpn*-DMR deletion can improve the developmental potential to E19.5; and strikingly, *Grb10*-DMR deletion allows pups to survive up to postnatal day 23 (Fig. 6a).

Interestingly, a recent study reported that AG-haESCs with the same maternal DMR deletions except *Xist*-DMR could be used to replace the oocyte genome to support bi-paternal development^45^. However, the birth rate of bi-paternal embryos was quite low (around 1% of transferred embryos) and all pups died a few days after birth. By contrast, incorporating *Xist*-DMR deletion in our strategy significantly increased birth rate of reconstructed embryos and improved postnatal development. This aligns with previous studies that *Xist* dosage balance is critical for embryonic development at mid-gestation^54^ and the failure of random XCI in the embryo proper also caused mice to be born at a lower frequency and smaller in size^55^. Additionally, SCNT studies have shown that the correction of skewed XCI pattern significantly improved the developmental efficiency of cloned embryos^32,33^. Taken together, these results indicate *Xist* dosage balance is critical for mid-gestation development, which is a foundation for later developmental stages.

Our data further demonstrated that DMRs with inverse parental origins can regulate embryonic development normally (Fig. 6b, c). This is consistent with reports showing that parental-origin inversion of gene expression in the *Dlk1*-*Dio3* locus can support normal development through generation of a mouse model carrying paternal deletion of IG-CGI and maternal deletion of IG-DMR^10^. Similarly, maternal expression of *Rasgrf1* or *Igf2*, normally expressed from the paternal allele, rescues the developmental abnormalities of mice with expression defects in *Rasgrf1* or *Igf2*^56,57^. Together, these findings suggest that the imprinted gene function based on differential expression pattern rather than parent-of-origin-specific manners. Importantly, these data support the assumption of “Conflict hypothesis”, which posits that genomic imprinting in mammals evolved to control the total expression levels of imprinted genes, with no qualitative differences between parental alleles^58^. However, how the current parental-origin pattern of the individual DMR is formed during evolution is an intriguing question^59–61^, which needs to be addressed in the future and may lead to the discovery of the origin of genomic imprinting.

Nevertheless, there are certain limitations in our Ha-Ha-fusion system. (1) Although 4N embryo complementation technology is a well-accepted strategy to test the in vivo developmental potential of injected pluripotent stem cells, the overall development potential of reconstructed embryos is limited due to the technical difficulties in ESC preparation and injection. We address this by using 4-cell 4N embryos instead of regular blastocysts as recipients, which improves the body chimeric proportion of fused ESCs and even germline transmission^62,63^. (2) Our strategy cannot analyze the role of imprinting in placental development. Nuclear transfer technology could address this limitation, but preliminary attempts showed that cloned embryos from XIK-DKO cells developed only to E10.5 (data not shown), in contrast to E13.5 of tetraploid complementation embryos with the same cells (Supplementary Fig. S2f). This result indicated that placental defects severely impaired the developmental potential of cloned embryos, which is consistent with our previous observation that defects in the trophoblast cell lineage account for the impaired in vivo development of cloned embryos^21^. In addition to DMR-regulated imprinted genes critical for placental development^22,64–71^, loss of H3K27me3 imprinting also disrupts post-implantation development of cloned embryos^72^. Rescuing the expression of H3K27me3-imprinted genes prevents the placental overgrowth in cloned animals^73,74^, highlighting the need to normalize H3K27me3-imprinted patterns for successful development of cloned embryos from Ha-Ha-fusion-derived cells. (3) CRISPR-Cas9-mediated DMR deletions may cause potential side effects, such as off-targets and even changes to the genome structure. To mitigate this, we analyzed at least 2 cell lines for each genotype and selected the cell lines with the best developmental potential for further analyses (Table 1). We also adopted the deletion strategy reported by previous references, in which the deleted DMRs have been proven to have the capacity to correct the monoallelic gene expression of the major genes in the imprinted cluster. Nevertheless, another strategy based on dCas9-mediated methylation rewriting reported by Wei et al. can avoid DNA deletions^75^, and thus might be very useful for future studies combined with the Ha-Ha-fusion system. (4) 8KO-DKO embryos develop to term efficiently. However, none of them survive to adult. This may result from DMR deletions failing to fully replicate physiological regulatory patterns (Fig. 3l, m; Supplementary Fig. S3g, h)^44^, or the absence of other critical imprinting loci in the Ha-Ha-fusion system. To address this, it is better to use haESCs from another genetic background such as JF1, which, based on SNPs, allows measuring the allele-specific expression of every imprinted gene in the genome after the different rescue attempts. This application can help us to better understand the changes in gene expression patterns after DMR deletions and probably reveal important clues that may be underlined in the lethality of 8KO-DKO embryos. Furthermore, this strategy can avoid the introduction of base mutations in the experiments of the DMR-switch between the paternal and maternal genome (Figs. 4h and 5f). In conclusion, while the Ha-Ha-fusion system provides a powerful platform for simultaneous manipulations of maternal and paternal imprints, further refinements are needed to enhance its efficiency and enable deeper exploration of imprinting network during development.

## Materials and methods

### Experimental mice

All animal procedures were performed under the ethical guidelines of Center for Excellence in Molecular and Cell Science (CEMCS), Chinese Academy of Sciences. All mice were housed in specific pathogen-free facilities of CEMCS. Female mice of BDF1 (C57BL/6×DBA/2) and ICR backgrounds were used to provide oocytes for micromanipulation and as pseudopregnant surrogates, respectively. *CAG-RFP* transgenic male mice (FVB/NJ background) were used to provide sperm for the establishment of androgenetic haploid ESCs. *Actin-EGFP* transgenic female mice (C57BL/6 background) were used to give oocytes for the establishment of parthenogenetic haploid ESCs.

### Derivation of haploid cell lines

Derivation of PG-haESCs was performed as previously described^19^. To derive GFP-labeled PG-haESCs, *Actin-EGFP* transgenic female mice (8–10 weeks) were induced to superovulate. The GFP-labeled oocytes were cultured in CZB medium for 1 h and then activated for 5–6 h in an activation medium containing 10 mM Sr^2+^. Following activation, embryos were cultured in KSOM medium with amino acids in an incubator with 5% CO_2_ at 37 °C. Similarly, the derivation of AG-haESCs was carried out, as previously described^12^. Embryos were collected at blastocyst stage and were transferred into ESC medium supplemented with 15% FBS, 1000 U/mL LIF, 3 M CHIR99021 (Stemcell Technologies, 100-1042), and 1 M PD0325901 (Stemcell Technologies, 100-0248). To sort haploid cells, ESCs were trypsinized and incubated with 15 mg/mL Hoechst 33342 in a 37 °C water bath. Subsequently, the haploid 1 C peak was purified using BD FACS AriaII for further culturing.

### CRISPR-Cas9-mediated imprinted region deletion in haploid cells

CRISPR-Cas9-mediated gene editing was performed as previously described^18^. To delete imprinted regions in haploid cells, sgRNAs were designed by sgRNA online design platform Chopchop (https://chopchop.cbu.uib.no) (Supplementary Table S1). Oligo DNAs were synthesized, and ligated to the pX330-mCherry (for GFP-PG-haESCs) or pX458-GFP (for RFP-AG-haESCs) plasmid (Addgene, 98750 and 48138). Plasmids were transfected into haploid ESCs by Lipofectamine 3000 (Invitrogen, L3000015). One day after transfection, the androgenetic and parthenogenetic haploid cells, respectively, expressing GFP and mCherry fluorescence protein were enriched by flow cytometry and plated at low density. Six days after plating, single colonies were picked for the derivation of haESCs. DMRs-deleted haploid cell lines were identified by DNA sequencing of PCR products amplified from targeted sites.

### Genotyping of ESC lines and reconstructed pups

Genotyping of ESC lines and reconstructed pups was performed by Sanger sequencing of PCR products amplified from targeted sites and examination using fluorescence microscopy. For DNA sequencing, two pair primers were designed to check deleted regions and WT regions respectively (Supplementary Table S2). After double-checking by gel electrophoresis, PCR products were performed by Sanger sequencing. For examination with fluorescence microscopy, cultured cells and tails of pups were examined by FITC and PE-Cy5 channels of Olympus IX 51 microscopy.

### Fusion of haploid cells to derive diploid cells

The AG- and PG-haploid cells, respectively, were suspended with 0.05% trypsin/EDTA and suspended in a fusion solution. Then two kinds of haploid cells were mixed and added with the Sendai virus (GenomONE, CF016EX). Mixture was incubated at 4 °C for 5 min and 37 °C for 15 min. Then mixture was plated in 6-well plate. After 48 h of plating, the diploid double positive (GFP and RFP) cells were purified by flow cytometry and plated at low density. 6 days after plating, single colonies were picked for the derivation of the diploid fused cells. The passage number of fused ESCs is inherited from the passage number of PG-haESCs.

### Derivation of tetraploid embryos

To generate fertilization-derived tetraploid embryos, B6D2F1 females were superovulated and mated with B6D2F1 males. Zygotes were collected from the oviduct 18–24 h after hCG injection. Two-cell embryos were obtained at 1.5 dpc. To generate tetraploid embryos, electrofusion was performed on two-cell embryos to produce one-cell tetraploid embryos. Two-cell embryos were aligned using an alternating current in 0.3 M mannitol solution, a single alternating current pulse of 6 V/cm was applied for 5 s, followed by a direct current pulse of 1000 V/cm for 20 µs. After electrofusion, the embryos were washed and cultured in KSOM.

### Tetraploid embryo complementation of ESCs

To generate tetraploid-complemented embryos, fused ES cells were fed with fresh medium 24–36 h before they were harvested for injection. Before injecting, ES cells were trypsinized, and suspended in HEPES-CZB medium. Previous studies have shown that 4-cell embryos could help ESCs exhibit high-proportion germline transmission^62,63^. Ten ESCs were microinjected into tetraploid 4-cell embryos to produce tetraploid-complemented embryos by blunt piezo-driven pipette. After culturing for one day, manipulated blastocysts were transferred into the uteri of the 2.5 dpc pseudopregnant mother. Recipient mothers carrying reconstructed embryos were euthanized at 19.5 dpc, and the pups were quickly removed from the uteri.

### Intracytoplasmic AG-haESC or sperm injection and embryo transfer

To generate SC embryos, AG-haESCs were treated with 0.05 μg/mL Demecolcine solution (Sigma) for 10–12 h and synchronized to the M phase. Next, the M-phase clones were treated with trypsin and suspended in the HCZB medium. MII oocytes were collected from oviducts of superovulated B6D2F1 females (8 weeks old). AGhaESCs were injected into the cytoplasm of MII oocytes in a droplet of HCZB medium containing 5 μg/mL cytochalasin B (Sigma, C6762) using a Piezo-drill micromanipulator. The injected oocytes were cultured in the CZB medium for 30 min and then activated for 5–6 h in Ca^2+^-free CZB with SrCl_2_. For sperm injection, the procedures were the same as described above. Following activation, the reconstructed embryos were cultured in AA-KSOM (Merk, MR-106) medium at 37 °C under 5% CO_2_ in the air. The embryos obtained through Intracytoplasmic AG-haESC Injection (ICAHCI) were cultured in KSOM medium for 24 h to reach the two-cell stage. Thereafter, 18–20 two-cell embryos were transferred into each oviduct of pseudopregnant ICR female mice at 0.5 dpc. Recipient mothers carrying SC embryos gave birth at 19.5 dpc.

### Bisulfite PCR

The genome extraction from ESCs used the TIANamp Genomic DNA Kit (TIANGEN, DP304-02). RNA was removed using RNaseA (ThermoFisher Scientific, R1253) in a 37 °C water bath for 1 h. DNA was recovered using a Universal DNA Purification Kit (TIANGEN, DP214-03). The bisulfite conversion was performed using the EZ DNA Methylation-Gold TM Kit (ZYMO research, D5005) for 200–500 ng genomic DNA, following the manufacturer’s instructions. The bisulfite DNA products were amplified by nested PCR. The amplified products were purified by gel electrophoresis using a Universal DNA Purification Kit and cloned into pMD™19-T Vector Cloning Kit (Takara, 6013). For each sample, more than 12 *E. coli* clones were picked for sequencing. The results were analyzed by the DNA methylation analysis platform. (http://services.ibc.unistuttgart.de/BDPC/BISMA/).

### Histological analysis

For histological analysis, adipose tissues from newborn pups were fixed in formaldehyde solution. Then the samples were dehydrated and embedded in paraffin. Sections were prepared with an ultramicrotome (Leica, RM 2235). Serial 5-µm sections were cut and collected on glass slides. The sections were stained with H&E after drying and photographed with microscopy (IX 51, Olympus).

### RNA extraction and real-time quantitative PCR (qPCR)

Total RNA was isolated from the cells using TRIzol reagent (Invitrogen, 15596018CN). The cDNA was obtained from about 1 μg RNA with a reverse transcription reaction by the HiScript III RT SuperMix for qPCR (Vazyme, R323-01). Real-time qPCR reactions (RT-qPCR) were performed on a Bio-Rad CFX96 using the ChamQ Universal SYBR qPCR Master Mix (Vazyme, Q711-02) in triplicate. All the gene expression levels were normalized to the internal standard gene Gapdh.

### WGBS

A total amount of 5–10 μg genomic DNA was mixed with 25 ng lambda DNA and sonicated to 200–500 bp, followed by end repair and dATP addition using the homemade Kit. Next, methylated adapters (synthesized by ThermoFisher Scientific) were ligated to the sonicated DNA. AMPure XP Beads (Vazyme, N411) were used to remove < 200 bp fragments. Bisulfite treatment was performed using the EZ DNA Methylation-Gold TM Kit (ZYMO research, D5005). After bisulfite conversion, the single-stranded, uracil-containing DNA was subjected to 10–12 cycles of PCR reaction with Illumina TruSeq PCR primers and 2.5 U of *Pfu* Turbo Cx Hotstart DNA polymerase (Agilent Technologies, 600410) to recover enough DNA for sequencing. The sequencing reads were aligned to mm9 using BSMAP with parameters –r 0 –w 100 –v 0.1 –A AGATCGGAAGAGC. Multiple mapped reads and PCR duplicates were removed. After mapping, those reads with total CG coverage less than 5 within 200 bp were removed. The methylation level was calculated using methylated CpG versus total CpG in each bin. The file with DNA methylation levels for each 200 bp bin was used for further analysis. For the boxplots about DNA methylation, we calculated the average DNA methylation levels of each region for each kind of gene element. DMR site information is presented in Supplementary Table S3.

## Supplementary information

Supplementary Information

## Data Availability

The raw sequence data reported in this manuscript have been deposited in the Genome Sequence Archive in the National Genomics Data Center, China National Center for Bioinformation/Beijing Institute of Genomics, Chinese Academy of Sciences (GSA: CRA021449, CRA021613) that are publicly accessible at https://ngdc.cncb.ac.cn/gsa.
