# Supplementary material for: Generation of live mice from haploid ESCs with germline-DMR deletions or switch
Source: Cell Discov. 2025 Jan 21;11:5. doi: 10.1038/s41421-024-00757-x (PMC11747502; doi:10.1038/s41421-024-00757-x)
Supplement: Supplementary file 1 — Supplementary Information [file 41421_2024_757_MOESM1_ESM.pdf]

Supplemental materials

Figure S1

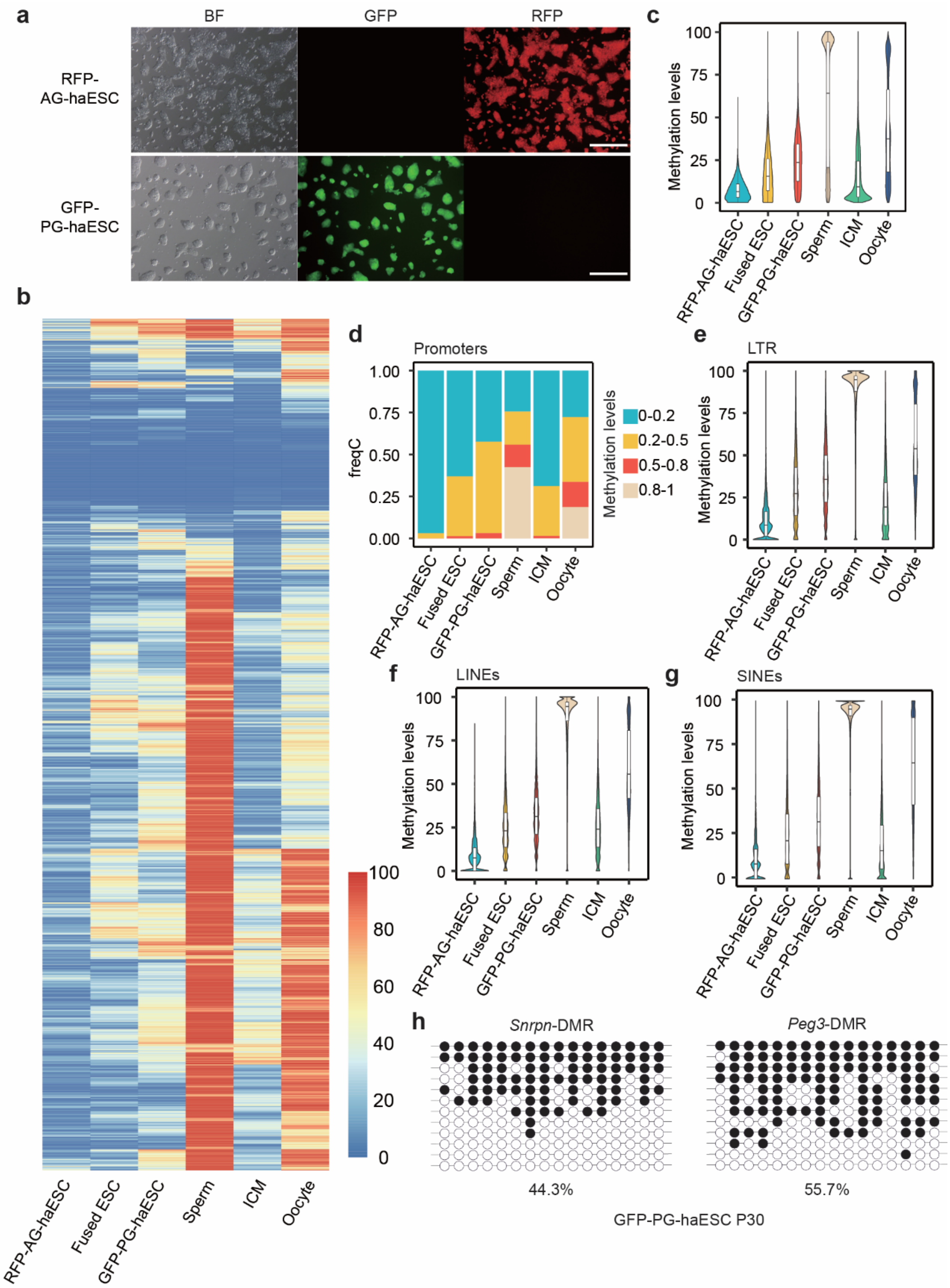

Supplementary Fig. S1 Haploid ESCs lost DNA methylation during long-term culture. a Colony morphology and fluorescence labeling of AG-haESCs and PG-haESCs. Scale bars, 200 um. BF, bright field. GFP, green fluorescent channel. RFP,

red fluorescent channel. **b** Heatmap showing the DNA methylation levels in oocytes, sperm, ICM, RFP-labeled AG-haESCs, GFP-labeled PG-haESCs and diploid fused ESCs. **c** Distribution of DNA methylation levels in all related samples. **d** Distribution of promoter DNA methylation levels in all related samples. **e**, **f**, **g** Methylation levels of LTR (**e**), LINES (**f**) and SINES (**g**) in all related samples. **h** Bisulfite sequencing analysis of *Snrpn*-DMR and *Peg3*-DMR in GFP-labeled PG-haESCs (passage 30). Open and filled circles represent unmethylated and methylated CpG sites, respectively.

Figure S2

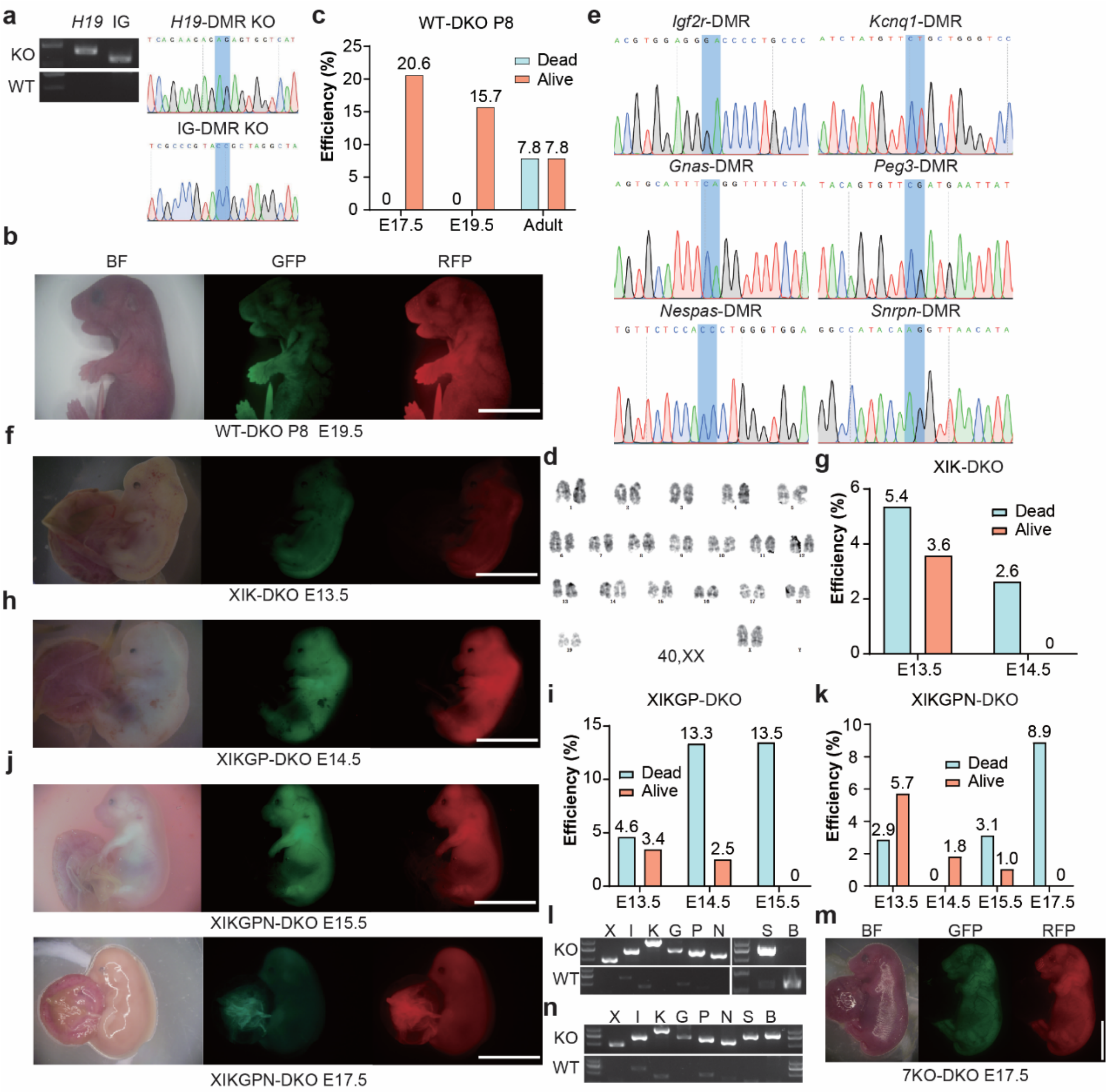

Supplementary Fig. S2 DMR deletions in GFP-PG-haESCs enhance developmental potential of reconstructed embryos. a Left, Genotyping of the RFP-DKO-AG-haESCs with PCR. Genotypes of the deletion and wild type at *H19*-DMR and IG-DMR were indicated on the top and bottom gel images. Right, DNA sequences of PCR products from Left. **b** E19.5 reconstructed embryos carrying paternal DKO derived from early passage (P8) WT-DKO fused cells. Scale bars, 5 mm. BF, bright field. GFP, green fluorescent channel. RFP, red fluorescent channel. **c** Development rates of WT-DKO P8 embryos at E17.5, E19.5 and adulthood. Alive status is judged by the heartbeat. Data came from Table1. **d** Karyotyping analysis of Fused ESCs. **e** DNA sequences of PCR products amplified from DMR-deletion regions of *Igf2r* (I), *Kcnq1* (K), *Gnas* (G), *Peg3* (P), *Nespas* (N) and *Snrpn* (S) locus. **f** E13.5 reconstructed embryos (XIK-DKO) carrying paternal DKO and maternal 3KO. 3 maternal regions are *Xist* (X), *Igf2r* (I) and *Kcnq1*(K). Scale bars, 5 mm. BF, bright field. GFP, green fluorescent channel. RFP, red fluorescent channel. **g** Development rates of XIK-DKO embryos at E13.5 and E14.5. Alive status is judged by the

heartbeat. Data came from Table1. **h** E14.5 reconstructed embryos (XIKGP-DKO) carrying paternal DKO and maternal 5KO. 5 maternal regions are *Xist* (X), *Igf2r* (I), *Kcnq1* (K), *Gnas* (G) and *Peg3* (P). Scale bars, 5 mm. **i** Development rates of XIKGP-DKO embryos at E13.5, E14.5 and E15.5. Alive status is judged by the heartbeat. Data came from Table1. **j** E15.5 (Top) and E17.5 (Bottom) reconstructed embryos (XIKGPN-DKO) carrying paternal DKO and maternal 6KO. 6 maternal regions are *Xist* (X), *Igf2r* (I), *Kcnq1* (K), *Gnas* (G), *Peg3* (P) and *Nespas* (N). Scale bars, 5 mm. **k** Development rates of XIKGPN-DKO embryos at E13.5, E14.5, E15.5 and E17.5. Alive status is judged by the heartbeat. Data came from Table1. **l** Genotyping of the GFP-XIKGPNS PG-haESCs with PCR. Genotypes of the deletion at *Xist* (X), *Igf2r* (I), *Kcnq1* (K), *Gnas* (G), *Peg3* (P), *Nespas* (N) and *Snrpn* (S) regions were indicated on the top gel images. Genotypes of the wild type at related regions were indicated on the bottom gel images. **m** E17.5 reconstructed embryos (7KO-DKO) carrying paternal DKO and maternal 7KO. Scale bars, 5 mm. **n** Genotyping of the GFP-XIKGPNSB PG-haESCs with PCR. Genotypes of the deletion at *Xist* (X), *Igf2r* (I), *Kcnq1* (K), *Gnas* (G), *Peg3* (P), *Nespas* (N), *Snrpn* (S) and *Grb10* (B) regions were indicated on the top gel images. Genotypes of the wild type at related regions were indicated on the bottom gel images.

Figure S3

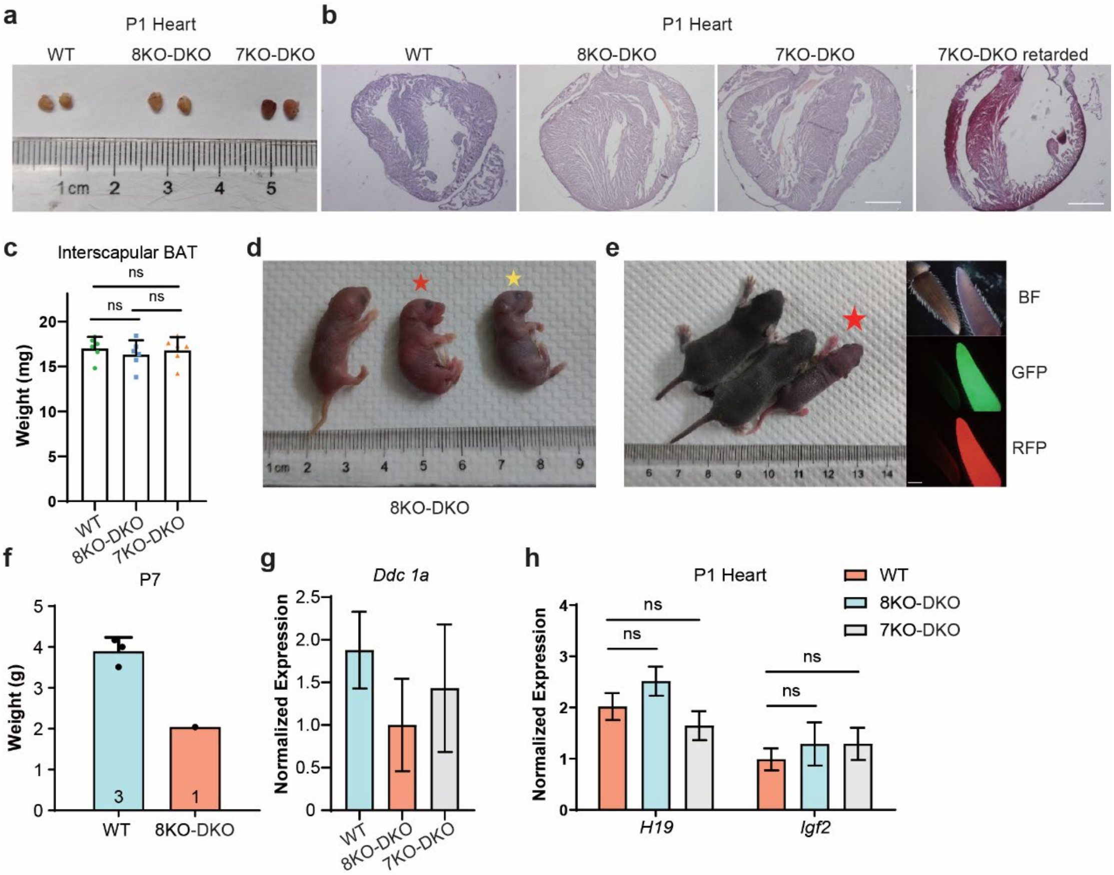

Supplementary Fig. S3 *Grb10*-DMR deletion further promotes developmental potential of reconstructed embryos from 7KO-DKO 2N fused ESCs. a Image of hearts from P1 BDF1 (WT), 8KO-DKO and 7KO-DKO mice. **b** H&E staining of hearts from P1 WT, 8KO-DKO and 7KO-DKO mice. Scale bars, 400 um. **c** The weights of iBATs from E19.5 wild-type (WT, n=6), 8KO-DKO (n=6) and 7KO-DKO (n=6) pups. **d** Postnatal 1day (P1) pups of WT and 8KO-DKO. Live 8KO-DKO pups were marked by red asterisk. Dead 8KO-DKO pups were marked by yellow asterisk. **e** Left, Postnatal 7 days (P7) mice of WT and 8KO-DKO. 8KO-DKO pup was marked by red asterisk. Right, Fluorescent images of 8KO-DKO pup tail. BF, bright field. GFP, green fluorescent channel. RFP, red fluorescent channel. **f** The body weights of P7 WT (n=3) and 8KO-DKO (n=1) mice. **g** Expression analysis of *Ddc 1a* in heart of P1 WT, 8KO-DKO and 7KO-DKO pups (n=3 for each group). **h** Expression analysis of *H19* and *Igf2* in heart of P1 WT, 8KO-DKO and 7KO-DKO pups (n=3 for each group). Data are presented as mean ± SD, analyzed by Student's t-test, ns, not significant.

Figure S4

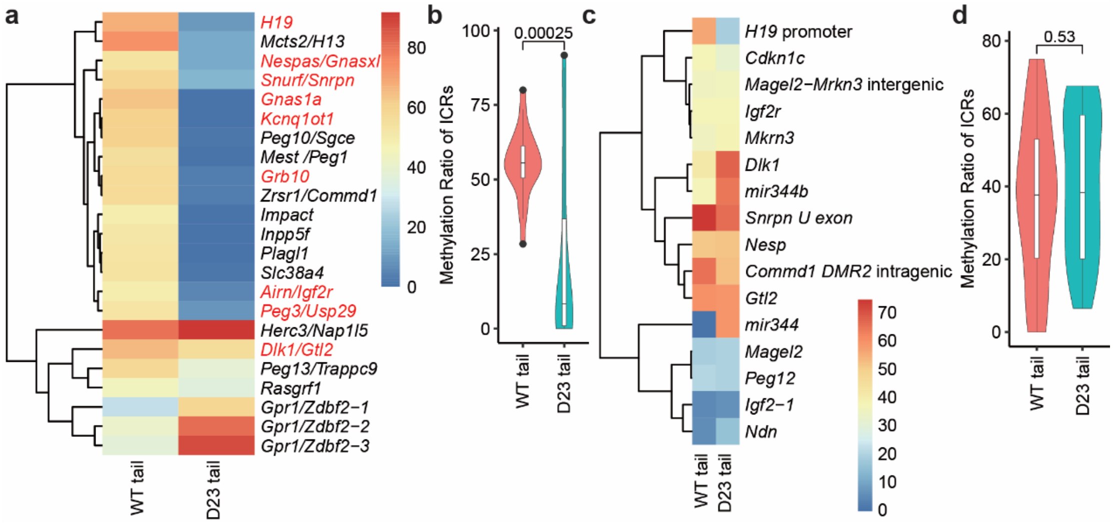

Supplementary Fig. S4 Methylation state of imprinted DMRs in wild-type (WT) and 8KO-DKO (D23) pups. a Heatmap of germline DMRs methylation in the tail of P23 BDF1 (WT) and 8KO-DKO (D23) mouse. Deleted DMRs were highlight with red color. **b** Distribution of DNA methylation levels at germline DMRs in the tail of WT and D23 mouse. **c** Heatmap of somatic DMRs methylation in the tail of WT and D23 mouse . **d** Distribution of DNA methylation levels at somatic DMRs in the tail of WT and D23 mouse. Data are presented as mean ± SD.

Figure S5

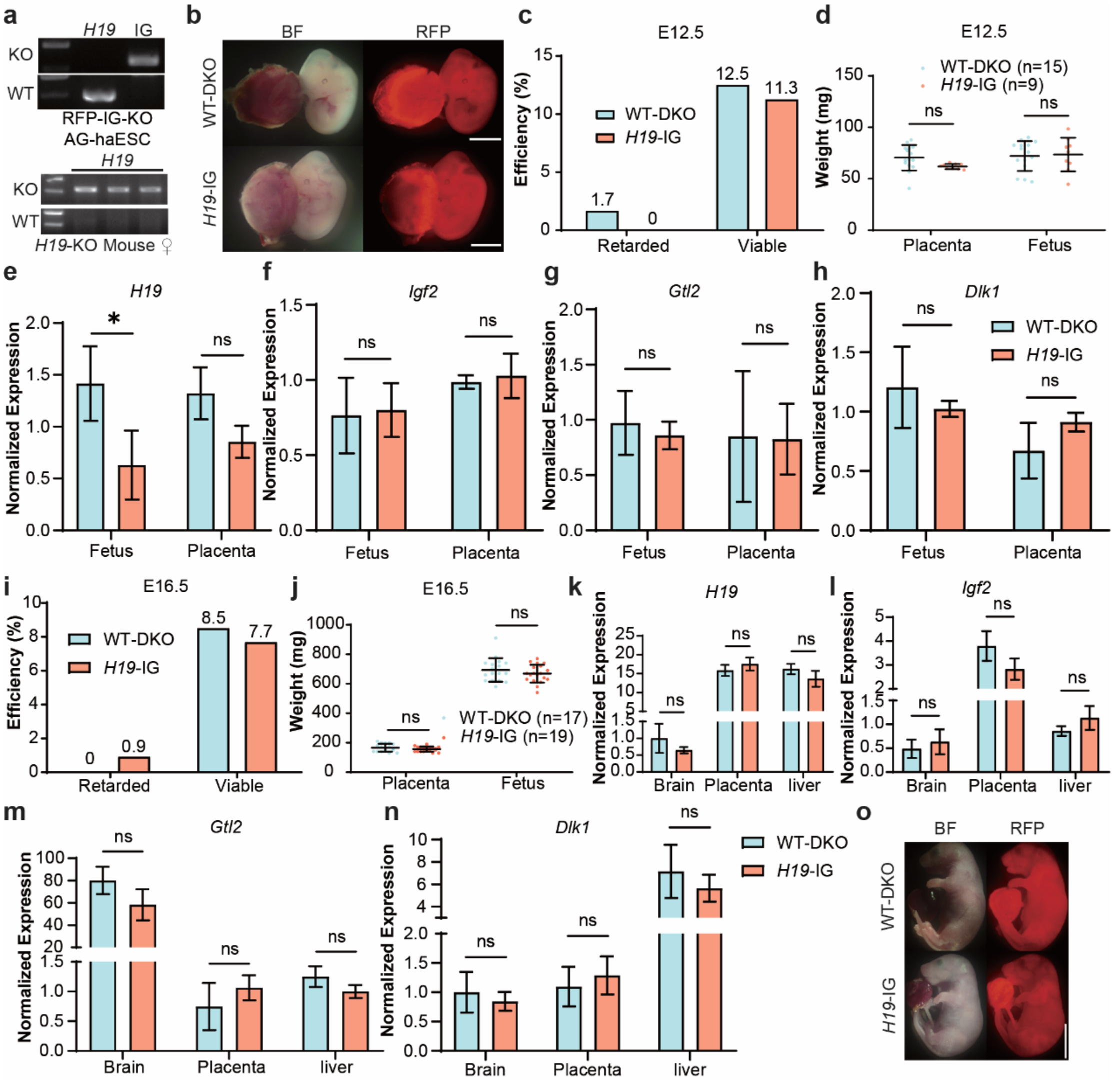

Supplementary Fig. S5 Maternal transmission of *H19*-DMR deletion equals to its paternal transmission. a Genotyping of the RFP-IG-KO AG-haESCs (top) and *H19*-DMR deletion mice (bottom) with PCR. Genotypes of the deletion and wild type at *H19*-DMR and IG-DMR were indicated on the top gel images. Genotypes of the deletion and wild type at *H19*-DMR were indicated on the bottom gel images. **b** Fluorescent images of E12.5 WT-DKO (top) and *H19*-IG (bottom) embryos obtained by ICAHCI. Scale bars, 5 mm. BF, bright field. RFP, red fluorescent channel. **c** A comparison of the development rates between E12.5 WT-DKO and *H19*-IG embryos. Viable status is judged by the heartbeat. Data came from Table2. **d** The fetal and placental weights of E12.5 WT-DKO (n=15) and *H19*-IG (n=9) embryos. Data are presented as mean ± SD, analyzed by Student's t-test, ns, not significant. **e**, **f**, **g**, **h** Expression analysis of *H19* (**e**), *Igf2* (**f**), *Gtl2* (**g**) and *Dlk1* (**h**) in fetus and placenta of E12.5 WT-DKO and *H19*-IG embryos (n=3 for each group). Data are presented as mean ± SD, analyzed by Student's t-test, $*P < 0.05$, ns, not significant. **i** A comparison of the development rates between E16.5 WT-DKO and *H19*-IG embryos. Viable status

is judged by the heartbeat. Data came from Table2. **j** The fetal and placental weights of E16.5 WT-DKO (n=17) and *H19*-IG (n=19) embryos. Data are presented as mean ± SD, analyzed by Student's t-test, ns, not significant. **k**, **l**, **m**, **n** Expression analysis of *H19* (**k**), *Igf2* (**l**), *Gtl2* (**m**) and *Dlk1* (**n**) in fetus and placenta of E16.5 WT-DKO and *H19*-IG embryos (n=3 for each group). Data are presented as mean ± SD, analyzed by Student's t-test, ns, not significant. **o** Fluorescent images of E19.5 WT-DKO (top) and *H19*-IG (bottom) embryos obtained by ICAHCI. Scale bars, 5 mm.

Supplementary Tables

Supplementary Table S1: List of sgRNA sequences in this study.

Primer Name	Sequence (5'-3')	Application
H19-DMR-sg1	CATGAACTCAGAAGAGACTGAGG	sgRNA sequence
H19-DMR-sg2	AGGTGAGAACCACTGCTGAGTGG	
IG-DMR-sg1	CGTACAGAGCTCCATGGCACAGG	
IG-DMR-sg2	CTGCTTAGAGGTACTACGCTAGG	
Xist-DMR-sg1	ATCTACCCCATGACTATTGCTGG	
Xist-DMR-sg2	CGGTCTCACCTTAGCACCATTGG	
Igf2r-DMR-sg1	ACATAATTTGGGAGTTGCCCAGG	
Igf2r-DMR-sg2	GGCAGGGGTGGTTAATAGCCTGG	
Kcnq1-DMR-sg1	GATTGTACGACCTAGCCTGCTGG	
Kcnq1-DMR-sg2	GACCTTCATCTATGTTCACCAGG	
Nespas-DMR-sg1	ATGGAGGGTGGTTCCGATAAAGG	
Nespas-DMR-sg2	TGACCGCGCTTAATTTCGCCTGG	
Gnas-DMR-sg1	GGGCTTCGAATAAGTTATTGTGG	
Gnas-DMR-sg2	AGTGGTCGCGCCAAGCGCAGAGG	
Peg3-DMR-sg1	TGGGAATAATTGATTACCGAGGG	
Peg3-DMR-sg2	AATTCATCGATTTTACTGCCAGG	
Snrpn-DMR-sg1	CTAGAGGTTATCGGTGTGTATGG	
Snrpn-DMR-sg2	TGTTAACCCCAGCTCAGGCTAGG	
Grb10-DMR-sg1	GCGTACTAACGGTTAGAAGAAGG	
Grb10-DMR-sg2	AATCTAGGGATGTCTAAGGATGG	

Supplementary Table S2: List of primer sequences in this study.

Primer Name	Sequence (5'-3')	Application
H19-BS-OF	GAGTATTTAGGAGGTATAAGAATT	Bisulfite sequencing
H19-BS-OR	ATCAAAAACTAACATAAACCCCT	
H19-BS-IF	GTAAGGAGATTATGTTTATTTTTGG	
H19-BS-IR	CCTCATTAATCCCATAACTAT	
IG-BS-OF	TTAAGGTATTTTTTATTGATAAAATA ATGTAGTTT	
IG-BS-OR	CCTACTCTATAATACCCTATATAATTA TACCATAA	
IG-BS-IF	GATCTCGAGCTCAAGCTTCGCTATAA TTTATCATAAACAAATCCCATAACTT ACT	
IG-BS-IR	CAGTTATCTAGATCCGGTGTTAGGAG TTAAGGAAAAGAAAGAAATAGTATA GT	
Snrpn-BS-OF	TATGTAATATGATATAGTTTAGAAAT TAG	
Snrpn-BS-OR	AATAAACCCAAATCTAAAATATTTTA ATC	
Snrpn-BS-IF	AATTTGTGTGATGTTTGTAATTATTTG G	
Snrpn-BS-IR	ATAAAATACACTTTCACTACTAAAAT CC	
Peg3-BS-OF	GTATTTAATTTGGAAAGTTGTAGGAG AG	
Peg3-BS-OR	TACAACAAAAATAAATCCCCCACCTC	
Peg3-BS-IF	GGAAAGTTGTAGGAGAGTAATTAAA	
Peg3-BS-IR	ACAAACTATTACTAAACACCCATTC	
H19-qPCR-F	CCTTCTTGAACACCATGGG	Realtime PCR
H19-qPCR-R	AGAGAAGTGTTAGCTCTTTGG	
Igf2-qPCR-F	CTTCTACTTCAGCAGGCCT	
Igf2-qPCR-R	CAGTATGTCTCCAGGAGGG	
Gtl2-qPCR-F	TCCTGGATTAGGCCAAAGC	
Gtl2-qPCR-R	AGCCTATTTGAGAAGCTGGT	
Dlk1-qPCR-F	CTGCGAAATAGACGTTCGG	
Dlk1-qPCR-R	GTACTGGCCTTTCTCCAGG	
Snrpn-qPCR-F	AAGATCAAGCCAAAGAATGC	
Snrpn-qPCR-R	CATTGAAACCAAGTTCTCCC	
Ndn-qPCR-R	GAGTACGAGTTCTTCTGGG	
Ndn-qPCR-R	CCATGATCTGCATCTTGGT	
Gapdh-qPCR-F	CACTCTTCCACCTTCGATGC	
Gapdh-qPCR-R	CTCTTGCTCAGTGTCCTTGC	
H19-DMR WT F	AGATGGGGTCATTCTTTTCC	Genotyping of *H19*-DMR WT
H19-DMR WT R	ATTGCTCTTAGCTTCTGTTG	
H19-DMR KO F	GTGGTTAGTTCTATATGGGG	Genotyping of *H19*-DMR knockout
H19-DMR KO R	TCTTACAGTCTGGTCTTGGT	

IG-DMR WT F	CTGGCACTCCGTTTCAGGAT	Genotyping of IG-DMR WT
IG-DMR WT R	TTCATTCATGGGGCCAGGTC	
IG-DMR KO F	TGTGCAGCAGCAAAGCTAAG	Genotyping of IG-DMR knockout
IG-DMR KO R	ATACGATACGGCAACCAACG	
Gnas-DMR WT F	TCTATCCGTGGTAGTCGGGT	Genotyping of *Gnas*-DMR WT
Gnas-DMR WT R	TCTTCAGAGGACCGACCCATA	
Gnas-DMR KO F	AAGCACAGCTGGAGTCTTCC	Genotyping of *Gnas*-DMR knockout
Gnas-DMR KO R	TGTGTCCACACACAAGCACT	
Nespas-DMR WT F	TGCATGGTGCATAATTGCCG	Genotyping of *Nespas*-DMR WT
Nespas-DMR WT R	ATTAAGCGCGGTCACAAAGC	
Nespas-DMR KO F	CCCCTCCCCGACTCTATGTA	Genotyping of *Nespas*-DMR knockout
Nespas-DMR KO R	CGCTTTCGTGCAAATAGGGG	
Xist--DMR WT F	TGGAGGTATCATCTCTCAAGTCTCC	Genotyping of *Xist*-DMR WT
Xist-DMR WT R	GGGCGATAGCACCCATGACA	
Xist-DMR KO F	ACTCATCCGCTTGCGTTCAT	Genotyping of *Xist*-DMR knockout
Xist-DMR KO R	ATCTGCCTGCTCACACAACT	
Kcnq1-DMR WT F	CCAGACCCGATTCGGTTTCA	Genotyping of *Kcnq1*-DMR WT
Kcnq1-DMR WT R	CGCGGGTTTCTTCTCTGAGT	
Kcnq1-DMR KO F	GGAGATGGCACACTCATACCA	Genotyping of *Kcnq1*-DMR knockout
Kcnq1-DMR KO R	AGAGCCAGGGGCATACTCAT	
Peg3-DMR WT F	GCATTCGTGCGGCAAACTAA	Genotyping of *Peg3*-DMR WT
Peg3-DMR WT R	GGCAGGGTCTTCGCAATCTA	
Peg3-DMR KO F	CCTGAAGGAGCACCTGAGTG	Genotyping of *Peg3*-DMR knockout
Peg3-DMR KO R	ATGGACCACGGTCTTGCATT	
Igf2r-DMR WT F	TCCACTCGAAATGCGCTCTG	Genotyping of *Igf2r*-DMR WT
Igf2r-DMR WT R	CCGAGGGTTCGGAGCAATTC	
Igf2r-DMR KO F	TTGGCAGCCCTCAGACAAAT	Genotyping of *Igf2r*-DMR knockout
Igf2r-DMR KO R	GGTTCTGGGTGGTAGTGACG	
Snrpn-DMR WT F	TTCTCCCCGTTCCCTACTGT	Genotyping of *Snrpn*-DMR WT
Snrpn-DMR WT R	AGACCCCTCCCCATTATGCT	
Snrpn-DMR KO F	AGCAAATTGTCTGCTTTAGCC	

Snrpn-DMR KO R	GGATCACCAGCTCCATTGAC	Genotyping of *Snrpn*-DMR knockout
Grb10-DMR WT F	ACGCACAGGACCATTACCAA	Genotyping of *Grb10*-DMR WT
Grb10-DMR WT R	CTGGGGACATTGCTGTCTGT	
Grb10-DMR KO F	TCTACAATGGCACGTTCACC	Genotyping of *Grb10*-DMR knockout
Grb10-DMR KO R	GCTGCCACTGTTTTTATCGC	

Supplementary Table S3: List of all maternal and paternal DMRs used in this study.

Name	Genome	MethAllele	Chromosome	Start	End
ZAC1-1	mm9	m	chr10	12810276	12810604
ZAC1-2	mm9	m	chr10	12810950	12811333
GRB10	mm9	m	chr11	11925485	11926335
COMMD1	mm9	m	chr11	22871842	22872319
PEG13/TRAPPC9	mm9	m	chr15	72636765	72642079
IGF2R/AIR	mm9	m	chr17	12934163	12935573
IMPACT	mm9	m	chr18	13130706	13132250
MCTS2	mm9	m	chr2	152512491	152513011
NNAT	mm9	m	chr2	157385786	157387398
NESPAS	mm9	m	chr2	174121208	174126482
GNAS	mm9	m	chr2	174152431	174154508
PEG10/SGCE	mm9	m	chr6	4697209	4697507
MEST	mm9	m	chr6	30686488	30689335
NAP1L5	mm9	m	chr6	58856690	58857056
PEG3/USP29	mm9	m	chr7	6680287	6684827
SNURF/SNRPN	mm9	m	chr7	67149878	67150301
NDN	mm9	m	chr7	69493100	69493581
INPP5F_V2	mm9	m	chr7	135831788	135832156
KCNQ1OT1	mm9	m	chr7	150481060	150481397
DLK1/DIO3	mm9	p	chr12	110761563	110768989
H19/IGF2	mm9	p	chr7	149766168	149768424
RASGRF1	mm9	p	chr9	89774406	89774691
